# Correction of the scimitar syndrome, a rare cardiac venous anomaly, leading to Budd–Chiari syndrome: a case report

**DOI:** 10.1186/1752-1947-8-273

**Published:** 2014-08-12

**Authors:** Marie-Pia Assoignon, Paul Christiaens, Wim Laleman

**Affiliations:** 1 Department of Gastro-enterology/Hepatology, University Hospital Gasthuisberg, Herestraat 49, 3000 Leuven, Belgium; 2 Department of Gastro-enterology, Bonheiden Imelda Hospital, Imeldalaan 9, 2820 Bonheiden, Belgium; 3 Department of Hepatology, University Hospital Gasthuisberg, Herestraat 49, 3000 Leuven, Belgium

**Keywords:** Budd–Chiari syndrome, Hepatic congestion, scimitar syndrome

## Abstract

**Introduction:**

Scimitar syndrome is a congenital heart disease characterized by an abnormal drainage of the right lung into the inferior vena cava, the right atrium or a variety of venous connections from the anomalous pulmonary vein to a systemic vein. This left-to-right shunt induces pulmonary hypertension and is an indication for surgical repair in cases of a history of recurrent pneumonia or significant left-to-right shunting. A corrective approach, which consists of rerouting the anomalous pulmonary flow to the left atrium, is usually performed. Complications of scimitar repair are stenosis, thrombosis and occlusion of the scimitar vein and its deviation.

**Case presentation:**

This case report describes a 53-year-old Caucasian woman with known scimitar syndrome, undergoing surgical repair due to invaliding symptoms of dyspnoea, and presenting with postoperative Budd–Chiari syndrome due to anomalous drainage of her right hepatic vein into the left atrium. It is an interesting cause of liver pathology caused by Budd–Chiari that never has been described before.

**Conclusions:**

This case report emphasizes the importance of a thorough preoperative evaluation, and the importance of antecedents in newly presenting pathology. It is an interesting cause of a known hepatic syndrome, the Budd–Chiari syndrome. This case report is of interest to many specialties, including Hepatology, Cardiology, Radiology and Cardiovascular Surgery. It exposes a new interesting anatomic variation of the scimitar syndrome with significant postoperative implications.

## Introduction

Scimitar syndrome is a rare congenital cardiovascular anomaly in which a part or the entire right lung is drained by pulmonary veins connecting to the vena cava, in combination with hypoplasia of the right lung, pulmonary hypertension and potential other cardiac defects [1-4]. Its name refers to the tubular opacity typically paralleling the right cardiac border that can be noticed on chest radiography and which resembles a curved Turkish sword or scimitar. The anomalous vein usually connects to the vena cava below the diaphragm, although many variations are described in which the scimitar vein drains to the junction between the inferior vena cava and the right atrium, straight to the right atrium, the coronary sinus or the hepatic or portal veins. Adult patients usually present with a history of repetitive pneumonia of the right lung, signs of pulmonary hypertension and/or chronic right ventricle volume overload [3-6].

This case report describes a 53-year-old Caucasian woman with known scimitar syndrome, undergoing surgical repair, and presenting with Budd–Chiari syndrome 1 month after surgery. In our case this abnormal drainage of the pulmonary vein to a hepatic vein eventually resulted in hepatic congestion and thus in Budd–Chiari syndrome. We have no knowledge of any other case presenting with this complication of scimitar syndrome.

## Case presentation

A 53-year-old Caucasian woman was diagnosed with scimitar syndrome a few years ago during routine chest radiography, showing the typical right-sided paracardial curvilinear projection, pathognomonic for the typical anomalous pulmonary venous drainage. The syndrome was subsequently confirmed on computed tomography (CT; Figure 1). During the following years, symptoms of fatigue and dyspnoea intensified correlating to significant left-to-right shunting, as demonstrated by magnetic resonance imaging (MRI). As a logical consequence, she underwent surgical repair by means of the long baffle technique in which a long intra-atrial tunnel is created with the right atrial wall as bottom and an artificial pericardial roof, connecting the origin of the scimitar vein to the left atrium [5-7]. Postoperatively, she improved significantly leading to discharge after 7 days. Unfortunately, 1 month later she presented at the emergency department with symptoms of abdominal bloating, belching and postprandial epigastric pain. Routine laboratory tests revealed mild inflammatory parameters and a discrete elevation of gamma-glutamyl transferase. A contrast-enhanced CT of her chest and abdomen showed a de novo hypertrophic left liver lobe and hypo-attenuation of her right liver lobe accompanied with a small amount of ascites in her pelvis. There was also a significant dilatation of her right hepatic vein. Close re-evaluation of the surgical region revealed an anomalous drainage of her right hepatic vein into the scimitar vein, instead of straight to the infradiaphragmatic vena cava in the usual presentation of the scimitar syndrome (Figure 2), therefore rerouting drainage of the right hepatic lobe to the left atrium. Unluckily, this impeded venous drainage of her right hepatic vein, and led to congestive hepatopathy or Budd–Chiari syndrome. The mechanism for this congestion is dual. First, the increased venous return to the left atrium resulted in an elevated pressure in the scimitar vein and left atrium, denying her right hepatic vein to drain. In addition, the surgical repair resulted in a 180-degree turnabout of the blood flow of the scimitar vein, as the pulmonary vein descends below the diaphragm, and is then routed upwards toward the left atrium, thus inducing a local diminished flow rate, enhancing venous congestion in her right liver lobe. Compensatory left lobe hypertrophy led to extrinsic compression of the stomach, which was partially responsible for her symptoms. There was no need for corrective surgery because she experienced no evidence of liver insufficiency or threatening portal hypertensive complications. As time went by, her left liver lobe, through hypertrophy, gradually took over the function of her right liver lobe which became progressively atrophic. A symptomatic medical treatment was established with dietary changes and gastroprokinetic drugs combined with a close follow up of the patient for more than a year.

**Figure 1:**
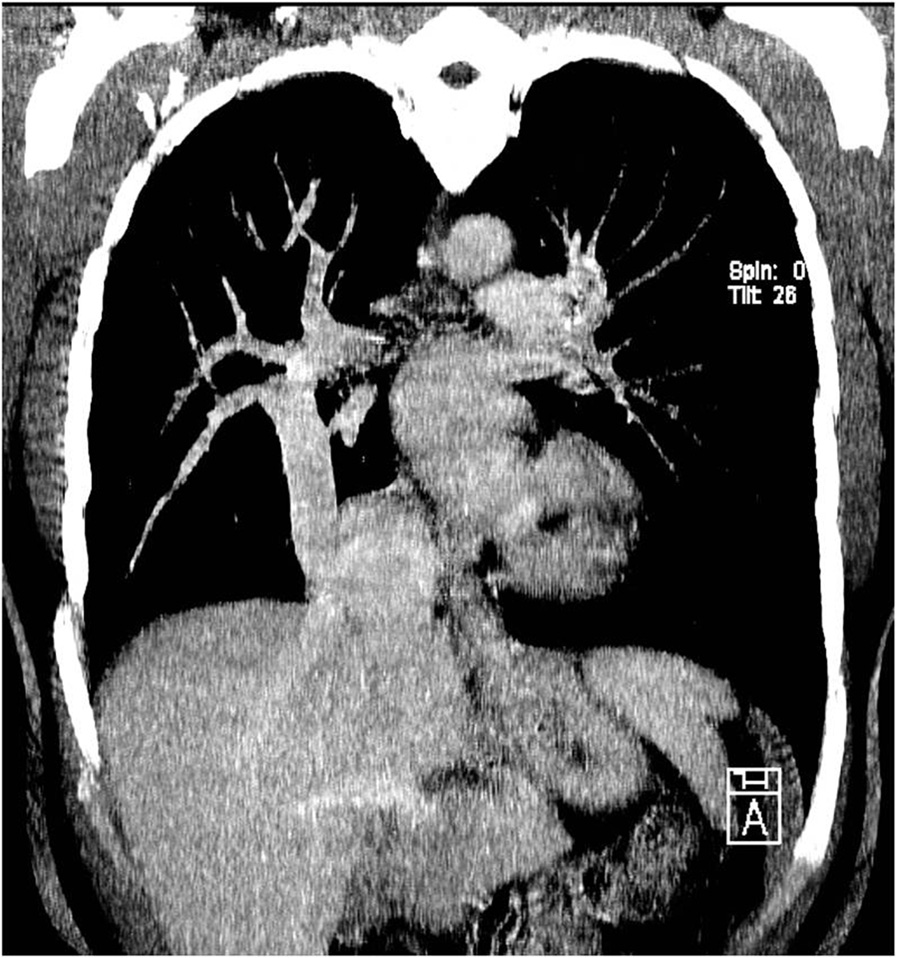
**Scimitar sign.** Coronal contrast-enhanced computed tomography shows the anomalous drainage of the right pulmonary vein into the inferior vena cava, representing the scimitar sign on conventional radiography.

**Figure 2:**
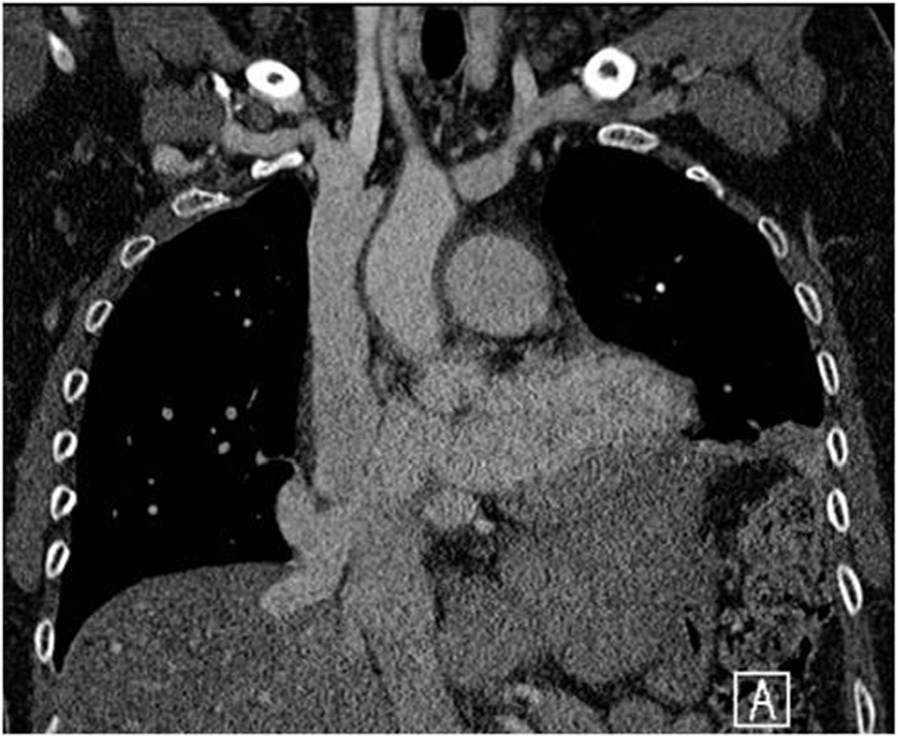
**Anomalous drainage of the right hepatic vein.** Coronal contrast-enhanced computed tomography scan shows the anomalous drainage of the right hepatic vein within the same orifice as the scimitar vein, redirecting the blood pool of the right hepatic vein into the left atrium. Note the normal drainage of the middle hepatic vein into the inferior vena cava.

## Discussion

Anomalous pulmonary venous return disorders are a specific group of congenital heart diseases caused by the abnormal drainage of a part or the entire right lung to the inferior vena cava, the right atrium or a variety of venous connections from the anomalous pulmonary vein to a systemic vein [4-6]. The estimated incidence is two out of 100,000 births [6]. When it is associated with hypoplasia of the right lung and pulmonary hypertension, it is specifically referred to as the scimitar syndrome. Agenesis of the hepatic vena cava with azygos or hemiazygos continuation to the vena cava superior and persistence of the hepatic venous are also described under this denominator [1,3,6,8].

In its infant form, the scimitar syndrome is diagnosed within the first 2 months after birth, with symptoms of failure to thrive, tachypnea, heart failure and cyanosis. There is an associated mortality of about 45%. When diagnosis is established beyond the neonatal period, patients are categorized into the paediatric or adult form with symptoms that are usually milder or even absent and depending on the degree of lung hypoplasia. Patients often have a history of repetitive pneumonia of the right lung, signs of pulmonary hypertension and/or chronic right ventricle volume overload [3,5,6].

The diagnosis, when considered, can easily be substantiated by a pathognomonic sign on chest X-ray, the scimitar sign, which is present in about 70% of the patients. The anatomical basis of this sign is the anomalous pulmonary vein descending below the diaphragm creating a half crescent sign at the right side of the heart and as such resembling a Turkish sword or scimitar. CT and MRI can both be used to delineate the exact anatomy of the anomalous drainage as well as for the additional assessment of concomitant congenital defects of the bronchovascular tree, lung or thoracic spine. Quantification of the severity of the left-to-right shunt can be assessed either through dynamic MRI or conventional cardiac catheterisation.

Surgical repair is nearly always required for the infantile form, while in the adult form it needs only to be considered in case of a history of recurrent right lung infections, significant left-to-right shunting or pulmonary hypertension [4-7]. The main aim of the procedures is to avoid sequelae of chronic heart overload. Procedures potentially to be considered are pneumectomy of the affected hypoplastic lung [5,6] or, more often applied, a corrective surgical intervention to reroute the anomalous pulmonary flow to the left atrium [5-7].

Complications of scimitar repair relate principally to stenosis, thrombosis and occlusion of the scimitar vein and its deviation. Anticoagulation and close follow up are essential [5,7].Our case represents an extremely rare complication. The orifice of the scimitar vein at the inferior vena cava was actually a common orifice with the right hepatic vein, as shown on multiplanar CT images (Figure 1). The pericardial patch, used during the long baffle procedure, was placed on this common orifice, therefore leading to anomalous connection of the right hepatic vein with the left atrium. The new anatomical condition with 180-degree turnabout of the blood flow in the scimitar vein, in combination with an elevated pressure in the left atrium and the scimitar vein due to an increased venous return to the left atrium, led to venous congestion, compensatory left liver hypertrophy and a small amount of ascites, comparable with a subacute presentation of Budd–Chiari syndrome.

Budd–Chiari syndrome is defined as a pathophysiological process resulting in an interruption or decrease of the normal blood flow from the liver into the vena cava [9]. It was first described in 1845 by George Budd [10,11] and has an incidence of 1 in 100,000, most frequently among women. A distinction is made between a primary and secondary form, depending on an intraluminal or extraluminal cause [9]. In the Caucasian population, thrombosis is the most frequent reason of vascular obstruction, whereas in Asian countries and in South Africa, membranous obstruction of the vena cava is more often the cause [10,12]. The pathophysiological mechanism is based on elevated sinusoidal pressure in the liver, secondary to the posthepatic obstruction, thus leading to sinusoidal congestion and hepatomegaly, possibly even to perisinusoidal necrosis of the hepatocytes and liver failure. Hepatomegaly and sinusoidal congestion result in hepatic pain, portal hypertension and ascites [10,11]. Depending on the severity of the obstruction, collateral drainage will develop through the lumbal veins, the vertebral venous plexus and the vena azygos and hemiazygos. Clinical presentation can be chronic, subacute or acute, depending on the development of collaterals. Acute onset is characterized by a triad of hepatomegaly, right hypochondrium tenderness and ascites. Collaterals hardly have time to develop. Elevated liver enzymes are witnessed, and a quick evolution to liver failure is to be feared [10]. In the subacute and chronic form, collaterals have more time to develop, and therefore symptoms are less severe, with insidious progression to portal hypertension and liver cirrhosis [10]. Diagnosis is made through ultrasound and CT, although MRI and arteriography give a more accurate image of the vascular anatomy. Therapy is based on retrospective studies and clinical experience, and the main goal is to achieve a decompression of the liver [9,11]. Medical therapy is often insufficient, and consists of diuretics to reduce ascites and a causative treatment of thrombosis. Angioplasty with balloon dilatation, stenting and transjugular intrahepatic portosystemic shunt offer a more adequate decongestion [9-11]. In some cases, transplantation is required.

Our patient presented with symptoms of subacute Budd–Chiari syndrome, including a vague abdominal discomfort, minimal liver function disorders, and minor ascites, established during a period of several weeks after surgery. As only a small part of her liver was affected, an evolution to liver failure was not to be expected. By contrast, her left liver lobe gradually became hypertrophic while her right became hypotrophic. Symptomatic treatment with dietary changes and gastroprokinetic drugs was enough to reduce her symptoms in combination with watchful waiting.

## Conclusions

In conclusion, we describe a rare anomalous pulmonary venous return disorder, the scimitar syndrome, complicated by a particular form of Budd–Chiari syndrome after surgical repair.

This case report emphasizes the importance of a thorough preoperative evaluation. Through CT and MRI the exact anatomy of the anomalous drainage and course of adjacent vessels should be delineated. It is an interesting cause of a known hepatic syndrome, the Budd–Chiari syndrome. This case report is of interest to many specialties, including Hepatology, Cardiology, Radiology and Cardiovascular Surgery. It exposes a new interesting anatomic variation of the scimitar syndrome with significant postoperative implications.

## Consent

Written informed consent was obtained from the patient for publication of this case report and accompanying images. A copy of the written consent is available for review by the Editor-in-Chief of this journal.

## Abbreviations

CT: Computed tomography; MRI: Magnetic resonance imaging.

## Competing interests

The authors declare that they have no competing interests.

## Authors’ contributions

MA analysed and interpreted the patient data and wrote the article, PC analysed and interpreted the patient data with regard to the hepatic presentation of the case. WL gave therapeutic advice and was a major contributor in writing the manuscript. All authors read and approved the final manuscript.
